# NLRP3, the inflammasome and COVID-19 infection

**DOI:** 10.1093/qjmed/hcad011

**Published:** 2023-01-20

**Authors:** Maureen Yin, Laura Marrone, Christian G Peace, Luke A J O’Neill

**Affiliations:** From the School of Biochemistry and Immunology, Trinity Biomedical Sciences Institute, Trinity College Dublin, Dublin, Ireland; CEINGE Biotecnologie Avanzate, Naples 80145, Italy; Dipartimento di Medicina Molecolare e Biotecnologie Mediche (DMMBM), “Federico II” University of Naples, Naples 80131, Italy; From the School of Biochemistry and Immunology, Trinity Biomedical Sciences Institute, Trinity College Dublin, Dublin, Ireland; From the School of Biochemistry and Immunology, Trinity Biomedical Sciences Institute, Trinity College Dublin, Dublin, Ireland

## Abstract

Severe coronavirus disease 2019 (COVID-19) is characterized by respiratory failure, shock or multiorgan dysfunction, often accompanied by systemic hyperinflammation and dysregulated cytokine release. These features are linked to the intense and rapid stimulation of the innate immune response. The NACHT, LRR and PYD domains-containing protein 3 (NLRP3) inflammasome is a central player in inflammatory macrophage activation which via caspase-1 activation leads to the release of the mature forms of the proinflammatory cytokines interleukin (IL)-1β and IL-18, and via cleavage of Gasdermin D pyroptosis, an inflammatory form of cell death. Here, we discuss the role of NLRP3 activation in COVID-19 and clinical trials currently underway to target NLRP3 to treat severe COVID-19.

## Introduction

Coronavirus disease 2019 (COVID-19) is an infectious disease caused by the Severe Acute Respiratory Syndrome Coronavirus 2 (SARS-CoV-2) virus. It has so far infected more than 640 million people worldwide and caused at least 6 million deaths. The most common symptoms of COVID-19 include fever, cough, fatigue, breathing difficulties and loss of smell or taste. One-third of people infected with SARS-CoV-2 are asymptomatic. Of those who present with symptoms, most people develop only mild-to-moderate symptoms, while 14% develop severe symptoms which include dyspnoea and hypoxia, and 5% have critical symptoms including respiratory failure, shock or multiorgan dysfunction. People with severe COVID-19 have symptoms of systemic hyperinflammation, mediated by a rapid release of inflammatory molecules, especially inflammatory cytokines such as interleukin (IL)-1β, IL-18, IL-6 and tumor necrosis factor-α, and the protein Gasdermin D (GSDMD) which is a marker of inflammatory cell death.^1^

The NACHT, LRR and PYD domains-containing protein 3 (NLRP3) inflammasome is a cytosolic signalling complex responsible for the secretion of the proinflammatory cytokines IL-1β, IL-18, and the induction of an inflammatory type of cell death called pyroptosis. NLRP3 activation has been positively correlated with COVID-19 disease severity and prognosis in the acute phase.^1–3^ In this review, we summarise recent findings on NLRP3 in COVID-19 during the acute phase of the disease and the therapeutic targeting of NLRP3.

## NLRP3 in COVID-19

**Figure 1.**
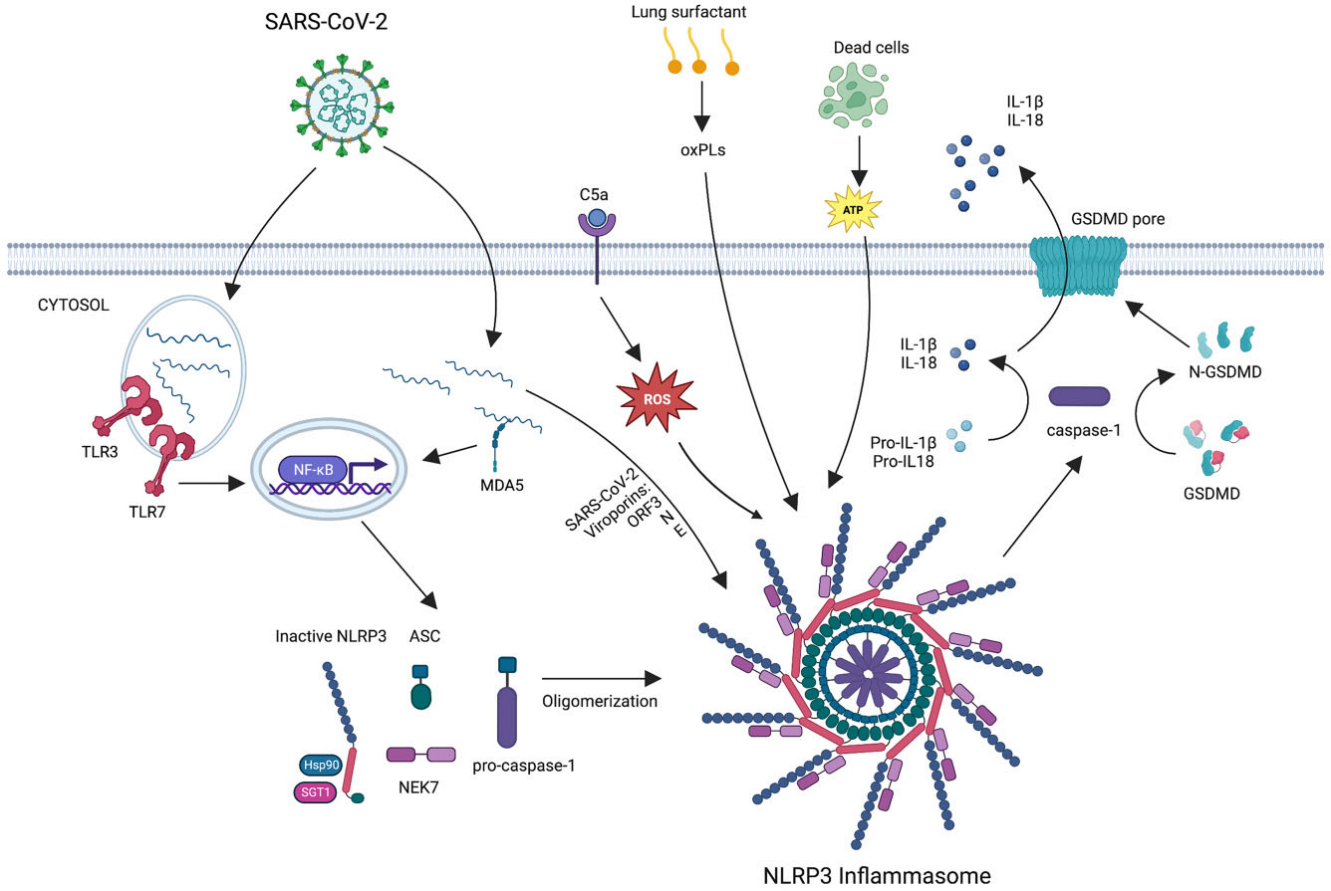
Mechanism of NLRP3 inflammasome activation in COVID-19. SARS-CoV-2-derived dsRNA and ssRNA can be sensed by endosomal TLR3 and TLR7, as well as by MDA5, which upregulate gene expression of components of NLRP3 and pro-forms of IL-1β and IL-18 via NF-κB. A second signal from the virus is required to oligomerize the NLRP3 inflammasome which then activates caspase-1 to cleave pro-IL-1β and pro-IL-18 into their mature forms. The NLRP3 may be activated by viroporins ORF3a, E, and N proteins, or host-intrinsic mechanisms such as complement protein C5a triggered ROS, oxidized phospholipids from lung surfactants, or ATP released from dead cells. IL-1β and IL-18 are then released from the cell through the GSDMD pore. Created with BioRender.com.

Inflammasomes are large inflammatory complexes mainly found in the cytosol of monocytes, macrophages and barrier epithelial cells that respond to pathogen-associated molecular patterns (PAMPs) or damage-associated molecular patterns (DAMPs).^4^ NLRP3 belongs to the NOD-like receptor (NLR) subfamily of Pattern Recognition Receptors (PRRs) that contain the pyrin domain and can be activated in most microbial infections, as well as by DAMPs and environmental irritants. NLRP3 together with the adaptor ASC protein PYCARD forms a caspase-1 activating complex known as the NLRP3 inflammasome (Figure 1). In the absence of activating signals, NLRP3 is in a complex with HSP90 and SGT1 in the cytoplasm. Recognition of PAMPs or DAMPs by PRRs such as Toll-Like Receptors (TLRs) primes the NLRP3 inflammasome by activating nuclear factor-κB (NF-κB) and induce the expression of the pro-forms of IL-1β and IL-18.^4^ A second activating signal is required to assemble and fully activate the inflammasome complex. Phagocytosed material such as uric acid crystals triggers the second signal, with efflux of potassium (K^+^) or chloride (Cl^−^) or calcium (Ca^2+^) influx as a common feature. In addition, ATP acting via P2X7 can also activate NLRP3 via potassium effluent.^5^ These events result in the release of HSP90 and SGT1 from NLRP3 and the recruitment of ASC and caspase-1. Caspase-1 is activated by proteolytic cleavage and in turn cleaves the pro-forms of IL-1β and IL-18, and GSDMD, which inserts itself into the membrane to form pores large enough to release both IL-1β and IL-18, and promotes pyroptosis, which is indicated by the release of lactate dehydrogenase (LDH).^4^

In COVID-19, upon entering cells via the surface protein angiotensin-converting enzyme 2 (ACE2), viral-derived dsRNA and ssRNA can be sensed by TLR3, TLR7 and melanoma differentiation-associated protein 5 (MDA5), which via NF-κB upregulate pro-IL-1β and pro-IL-18 that are later cleaved into their active forms by fully activated NLRP3 (Figure 1). Upon translation of the viral proteins, the viroporins open reading frame 3a and the envelope (E) can cause K^+^ efflux or Ca^2+^ influx to activate the NLRP3 inflammasome.^6^ Viral N protein can also bind to NLRP3, resulting in a direct activation.^7^ NLRP3 is also proposed to be activated by a range of host-intrinsic mechanisms, for example, oxidized phospholipids from oxidation of lung surfactant phospholipids,^8^^,^^9^ reactive oxygen species (ROS) triggered through the binding of complement protein C5a to the host surface C5aR1 receptor,^1^^,^^10^ and ATP released from dead cells.^1^

An early indication of a role for NLRP3 in COVID-19 came from an association between LDH levels and several disease severity scores.^11^ This was later confirmed in other cohorts.^12–14^ LDH release might be a consequence of the cell death seen in the lungs and kidneys in COVID-19.^1^^,^^15^^,^^16^ Rodrigues and colleagues then provided evidence for the role of NLRP3 in COVID-19 by demonstrating its activation in PBMCs from COVID-19 patients.^2^ Cleaved caspase-1 and IL-18 were detected in the sera of COVID-19 patients and were positively correlated with disease severity, poor prognosis and other COVID-19 severity serum markers, including IL-6 and LDH. IL-1β induces the production of IL-6, another abundantly detected cytokine in severe COVID-19 patients.^17^^,^^18^ IL-6 stimulates the release of various acute phase proteins, such as the hepatic factors C-reactive protein and ferritin, which are associated with poor prognosis.^19–21^ IL-18 has been linked to ferritin production^22^ and is another line of evidence for NLRP3 in severe COVID, as its level is significantly higher in symptomatic patients and is increased in accordance with disease severity.^23^ IL-1β itself has been associated with severe COVID-19.^24^ At the start of the pandemic in early 2020, increased IL-1β in patient sera was already observed in Wuhan patients infected with SARS-CoV-2.^25^ A longitudinal study that profiled patient cytokine changes detected increased IL-1β and IL-18 correlating with disease severity.^26^ Finally, the IL-1 receptor antagonist (IL-1Ra) which blocks IL-1 has also been associated with severe COVID-19.^26–28^

GSDMD has also been consistently observed in the serum of COVID-19 patients.^2^^,^^29–31^ High expression of GSDMD is also associated with the release of neutrophil extracellular traps (NETs), a phenomenon associated with immunocoagulopathy, and organ damage found in severe COVID-19 cases.^32^ Higher serum GSDMD levels have been correlated with the requirement for mechanical ventilation and areas of consolidation (defined as opacities that conceal the underlying vessels and are associated with disease severity) on lung CT in COVID-19 patients, suggesting pyroptosis in the disease manifestations.^33^

NLRP3 is activated to limit the infection by SARS-CoV-2 as its inhibition with the small molecule MCC950 led to the release of the virus by infected macrophages,^34^ presumably via inhibition of pyroptosis, since that would sustain the macrophage to allow for more viral replication.

## Pharmacological blockade of NLRP3 in COVID-19

NLRP3 is linked to many inflammatory conditions, including atherosclerosis, Alzheimer’s disease and inflammatory bowel disease, and many drugs used in treating these diseases are being repurposed in the treatments for COVID-19.^1^ Several IL-1 signalling inhibitors have been studied for their effectiveness against COVID-19, including a human IL-1RA (also known as anakinra), a soluble decoy receptor and a neutralizing monoclonal antibody.^35^

Several observational studies have shown promising results of anakinra in reducing CRP and the need for invasive mechanical ventilation (Table 1).^36–45^ Anakinra has also been tested in randomized control trials (RCTs) but received mixed results.^53–56^ In the SAVE-MORE double-blinded trial, 594 patients with the risk of progressing to respiratory failure were provided with anakinra or a placebo, and 86.9% of the patients also received dexamethasone. Of the patients who received anakinra, 50.4% showed complete recovery with no viral RNA detected after 28 days, compared with only 26.5% in the placebo group.^56^ Anakinra was also independently associated with clinical benefit at day 14, and reduced risk of persistent disease at day 28. Despite the success of the SAVE-MORE trial, two later RCTs failed to detect any difference between the anakinra and placebo group in patients with moderate-to-severe disease.^53^^,^^54^ On the other hand, another smaller, not blinded trial CORIMUNO-ANA-1 was stopped early due to a lack of significant reduction in the need for ventilation or mortality.^55^ The results concluded that anakinra did not improve outcomes in patients with mild-to-moderate COVID-19 pneumonia. Anakinra might be beneficial in patients with moderate-to-severe disease, and at risk for progression to respiratory failure, but less effective in patients already suffering from respiratory failure.^57^ Also, corticosteroids combined with anakinra appear to improve clinical outcomes better than anakinra alone.^56^

**Table 1.** NLRP3 inflammasome-targeting therapeutics in COVID-19

Drug	Tested clinically	Main findings
Anakinra	Yes	Decreased viral load after 28 days, decreased severity at day 14, and reduced risk of persistent disease at day 28. In patients with moderate-to-severe disease, Anakinra lacked efficacy.^36–45^
MCC950	No	Inhibited caspase-1 activation and IL-1β production in *in vitro* SARS-CoV-2 infection of primary human monocytes and reversed lung pathology in a mouse model of infection but increased the release of virus from macrophages.^2^^,^^34^
Glyburide	No	Reduced SARS-CoV-2-driven monocyte lytic death, caspase-1 activation, IL-1β and IL-6 production in human monocytes.^30^
DFV890	Yes	One trial found earlier viral clearance, however, very modest effects were observed in terms of disease severity and disease outcome.^46^
Colchicine	Yes	Some studies have shown a decreased risk of mortality and rate of intubation and increased discharge rate. However, some drug regimens failed to show an effect. Adverse events included skin rash and diarrhea.^47–52^
Dapansutrile	Yes	Trial underway

Of note, 11 patients were reported with bacterial and fungal sepsis compared to only four in the usual care group, which is likely associated with immunosuppression caused by broad IL-1 targeted therapy.^55^ Specific NLRP3 targeting might allow the production of IL-1β by other inflammasomes and reduce the risk of infections associated with the use of anakinra which will block IL-1 driven by any inflammasome.^35^ MCC950 is a potent and selective inhibitor for the NLRP3 inflammasome but does not inhibit other inflammasomes such as AIM2, NLRC4 or NLRP1.^58^ NLRP3 inhibition by MCC950 reduced cytokine production and lung cellular infiltrates in influenza A virus infection in mice, a type of infection that bears many similarities to that of SARS-CoV-2.^59^ MCC950 was also shown to inhibit caspase-1 activation and IL-1β production in an *in vitro* SARS-CoV-2 infection of primary human monocytes.^2^ Importantly, MCC950 also reversed chronic lung pathology in a mouse model of SARS-CoV-2 infection.^34^ That study also demonstrated that NLRP3 is activated to limit the infection by SARS-CoV-2 as its inhibition with MCC950 led to the release of the virus by infected macrophages,^34^ presumably via inhibition of pyroptosis, since that would sustain the macrophage to allow for more viral replication. This might mean that inhibition of NLRP3 while having an anti-inflammatory effect might increase viral replication which could have unwanted consequences.

Another NLRP3 inhibitor glyburide reduced SARS-CoV-2-driven monocyte lytic death, caspase-1 activation, IL-1β and IL-6 production in an *in vitro* model using human monocytes.^30^ Two phase II clinical trials are testing direct inhibition of NLRP3 in patients (Novartis, NCT0432053; Olatec Therapeutics, NCT04540120). The Novartis trial utilized a specific NLRP3 inhibitor DVF890 in a total of 143 participants with mild-to-moderate COVID-19.^46^ DFV890 demonstrated subtle improvement in viral clearance on day 7, clinical status and mortality, but failed to significantly improve the combined APACHE II score compared to those who received standard of care.^46^ The Olatec trial uses another specific inhibitor of NLRP3, dapansutrile, and aims to access the safety and efficacy of a NLRP3 inhibitor in patients with moderate symptoms. The outcome of the Olatec trial has yet to come out.

Indirect NLRP3 inhibitors have also been tested in the clinic, for example, colchicine, which is currently used to treat gout and Adamantiades-Behçet’s disease.^60^ In addition to interfering with monocyte and neutrophil chemotaxis, colchicine also indirectly inhibits NLRP3 activation.^61^ Indeed, colchicine has been tested in multiple RCTs for COVID-19.^47–52^ The RECOVERY (Randomised Evaluation of COVID-19 Therapy) arm with colchicine was stopped due to futility.^49^ Like the other NLRP3-targeted drugs mentioned above, colchicine seemed to have minimal effects in community-treated patients without a mandatory diagnostic test but led to a lower rate of the composite of death or admission to the hospital among those with PCR-confirmed COVID-19.^50^ In a small trial, colchicine demonstrated a reduction in the length of both supplemental oxygen therapy and hospitalization in patients with moderate-to-severe COVID-19.^52^ Beneficial effects were also observed in other trials where colchicine statistically significantly improved time to clinical deterioration^51^ and clinical condition.^48^

## Conclusion

Since the beginning of the pandemic, there have been numerous attempts to develop therapeutics for COVID-19, and NLRP3 has been an attractive target. The pre-clinical evidence for NLRP3 in COVID-19, the correlation with outputs from NLRP3 and disease severity, the partial success of anakinra in trials, and a marginal benefit provided by the NLRP3 inhibitor indicate that further studies are warranted into the targeting of NLRP3, most likely in stratified trials, to limit the damaging effects of inflammation occurring during COVID-19.

## Supplementary Material

hcad011_Supplementary_Data
